# Microglial TAK1 promotes neurotoxic astrocytes and cognitive impairment in LPS-induced hippocampal neuroinflammation

**DOI:** 10.1016/j.jbc.2025.110225

**Published:** 2025-05-09

**Authors:** Xiao Han, Xin Cao, Qianqian Ju, Chengxin Ge, Yongqi Lin, Jinhong Shi, Xinhua Zhang, Cheng Sun, Haoming Li

**Affiliations:** 1 Department of Human Anatomy, Medical School of Nantong University, Nantong, Jiangsu, China; 2 Key Laboratory of Neuroregeneration of Jiangsu and Ministry of Education, Co-Innovation Center of Neuroregeneration, Nantong University, Nantong, Jiangsu, China

**Keywords:** TAK1, microglia, astrocyte, hippocampus, neuroinflammation

## Abstract

The peripheral immune system has a strong effect on the central nervous system (CNS). Systemic lipopolysaccharides (LPS) administration triggers robust microglial activation and induces significant inflammatory responses in the hippocampus. This study investigates the role of Transforming Growth Factor-β-Activated Kinase 1 (TAK1) in mediating LPS-induced hippocampal neuroinflammation and cognitive impairment. Our findings reveal that LPS induces activation of microglial TAK1, which in turn activates downstream effector NF-κB/p65 to release pro-inflammatory cytokines. The activated microglia also promote astrocytes to polarize into a neurotoxic phenotype (A1-like phenotype) and cause the loss of newborn neurons in the hippocampal dentate gyrus (DG). However, TAK1 reduction inhibits microglial responses, limits neurotoxic astrocytes, rescues newborn neurons, and subsequently improves LPS-induced cognitive deficits, suggesting that targeting TAK1 may be an effective strategy for alleviating neuroinflammation. The interaction between TAK1 activation, microglial responses, and the transition of neurotoxic astrocytes enhances our understanding of the cellular dynamics driving LPS-induced neuroinflammation, suggesting that TAK1 may be a therapeutic target for treating cognitive impairment.

Microglia, the resident immune cells of the central nervous system (CNS), maintain homeostasis and respond to injury or disease. These cells exhibit dynamic states influenced by local CNS signals, differing in morphology, transcriptional, and functional profiles across brain regions. The functional and transcriptional diversity of microglia is revealed by recent advances in single-cell RNA sequencing (scRNA-seq) and spatial transcriptomics (1). Microglia are involved in tissue repair and the clearance of toxic aggregates, such as amyloid plaques and tau tangles, which are associated with Alzheimer's disease (AD) and Parkinson's disease (PD) (2, 3, 4, 5). Accumulating evidence suggests that disease-associated microglia (DAM), a recently identified subset of microglia, might also play a protective role in AD (6, 7, 8). Thus, targeting microglial activation could mitigate the progression of neurodegenerative diseases (9).

The peripheral immune system has a strong effect on CNS. Systemic lipopolysaccharides (LPS) administration can trigger robust microglial activation and lead to the release of a variety of inflammatory cytokines, mainly *via* NF-κB signaling (10, 11, 12). This activation process is essential for understanding the mechanisms underlying neuroinflammation. The neuroprotective effects of certain compounds, such as Hirsutine from *Uncaria rhynchophylla* or Saikosaponins B2 from *Radix Bupleuri*, have been shown to inhibit LPS-induced microglial activation and neurotoxicity (13, 14), suggesting that modulating microglial activity could serve as a therapeutic target to alleviate inflammatory responses.

Recently, the interaction between microglial activation and astrocyte polarization has been recognized as a critical aspect of neuroinflammation (15). Research shows that activated microglia causes astrocytes to polarize into an A1-like phenotype (16). This transition to a neurotoxic phenotype involves increased levels of pro-inflammatory cytokines and neurotoxic factors, which contribute to neuronal degeneration and impair cognitive function. Blocking microglial activation may help reduce harmful effects by preventing the transition of astrocytes into an A1-like state (17, 18, 19). Therefore, targeting microglial activation and modulating astrocyte polarization could be a reliable approach to alleviate inflammatory responses in the CNS.

Transforming growth factor-β-activated kinase 1 (TAK1), encoded by the Mitogen-activated protein kinase kinase kinase 7 (*Map3k7*) gene, is an essential signaling molecule found in microglia (20, 21). TAK1 serves as a pivotal mediator in the signaling cascades of pro-inflammatory cytokines, which in turn affect NF-κB and MAPK signaling pathways (22, 23). TAK1 inhibition significantly decreases MAPK activation and subsequent inflammatory responses in microglia, potentially providing protective effects against neuronal degeneration (24, 25, 26). However, the specific contributions of TAK1 to the cross-talk between microglia and astrocytes in the context of LPS-induced neuroinflammation have not been thoroughly investigated.

This study aims to investigate the activation of microglia in response to systemic LPS administration and the subsequent effects on astrocyte activation and neurotoxicity. To achieve this, we used both *in vivo* mouse models and *in vitro* cell culture to clarify the essential function of TAK1 in this process. Our data demonstrate that microglial TAK1 is crucial for the transition of astrocytes to a neurotoxic state and subsequent neuroinflammatory responses.

## Results

### Systemic LPS administration activates TAK1 in hippocampal microglia

**Figure 1:**
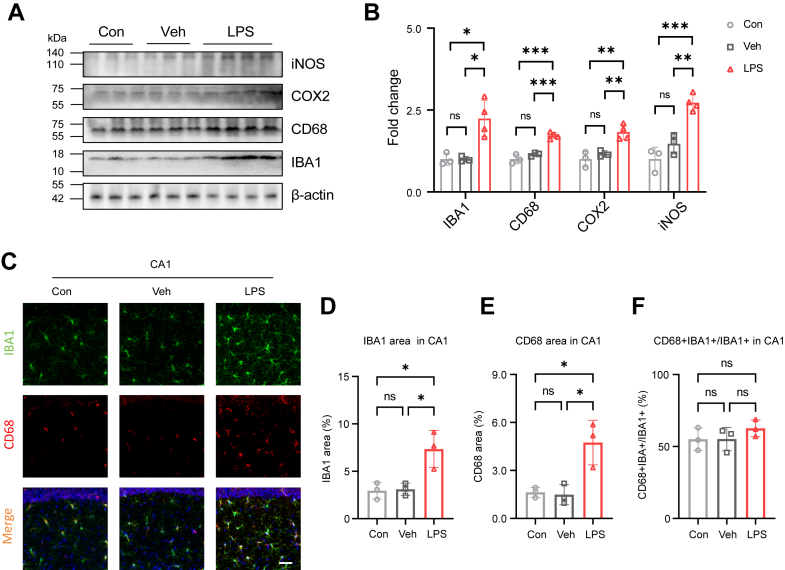
**LPS intraperitoneal (i.p.) administration activates microglia in the hippocampus.***A*, immunoblot analyses of IBA1, CD68, COX2, and iNOS in the uninjected control group (Con), vehicle group (Veh), and LPS group (n = 3 in Con and Veh, n = 4 in LPS). *B*, quantification of IBA1, CD68, COX2, and iNOS levels. *C*, representative images of IBA (*green*) and CD68 (*red*) immunostaining in hippocampal CA1 (n = 3 in each group). Scale bar=50 μm. *D*, quantification of IBA1 immunoreactivity area in CA1. *E*, quantification of CD68 immunoreactivity area in CA1. *F*, quantification of CD68+IBA+ cells in IBA1+ microglia in CA1. One-way ANOVA followed by Tukey’s multiple-comparisons (*B*, *D*–*F*) or the Brown-Forsythe and Welch ANOVA test (*B*). ∗*p* < 0.05, ∗∗*p* < 0.01, ∗∗∗*p* < 0.001, ns, non-significant.

Systemic inflammation has emerged as a common feature across dysfunctions of various organs. In the CNS, systemic LPS administration induces significant microglial activation, characterized by increased expression of pro-inflammatory cytokines (10). In this study, we first examined microglial activation by LPS at different time points under our experimental conditions in the hippocampus, a region critically involved in cognitive functions and often affected by neuroinflammation (27). An LPS dose of 0.5 mg/kg is approximately 20-fold lower than the lethal dose and is often used in research to explore the link between the brain and the immune response to infection (28, 29, 30). After treatment with 0.5 mg/kg LPS intraperitoneally (i.p.) for seven consecutive days (Fig. S1*A*), the protein levels of IBA1 and CD68 increased (Fig. 1, *A* and *B*; Fig. S1, *B* and *C*). Cyclooxygenase 2 (COX2) and inducible nitric oxide synthase (iNOS) act as markers of the inflammatory response, facilitating the production of pro-inflammatory cytokines and mediators that can exacerbate neuronal degeneration (31, 32). LPS significantly increased the levels of these factors (Fig. 1, *A* and *B*; Fig. S1, *B* and *C*). IBA1, CD68, COX2, and iNOS levels in vehicle group (Veh) were not significantly changed compared to uninjected control group (Con) (Fig. 1, *A* and *B*). CD68 is indicative of the microglial response to various pathological conditions, including neurodegenerative diseases and infections (33, 34). We found that IBA1 and CD68 positive areas in LPS group were elevated in hippocampal cornu ammonis (CA) 1 region (Fig. 1, *C*–*E*). The CD68 was exclusively co-localized with IBA1, but the percentage of CD68+IBA+ cells in microglia was not significantly increased in the LPS group (Fig. 1*F*). Similar results were found in the hippocampal CA3 (Fig. S1, *D*–G) and DG (Fig. S1, *H*–*K*). Overall, the increased levels of IBA1, CD68, COX2, and iNOS reflect microglial activation and neuroinflammation, both of which aggravate the progression of pathology in hippocampus.

**Figure 2:**
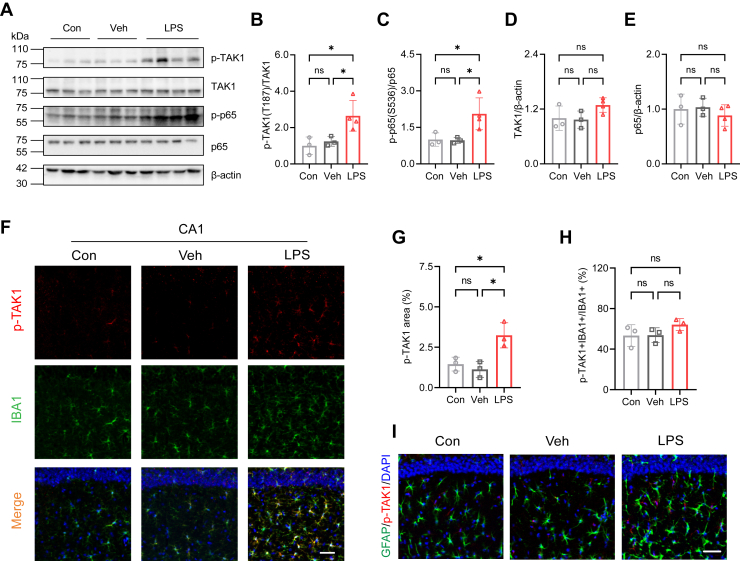
**LPS administration induces microglial p-TAK1 activation in the hippocampus.***A*, immunoblot analyses of p-TAK1 (T187), TAK1, P-P65 (S536), p65 in Con, Veh, and LPS groups (n = 3 in Con and Veh, n = 4 in LPS). *B*, quantification of p-TAK1 (T187) after LPS administration. *C*, quantification of p-p65 (S536) after LPS administration. *D* and *E*, quantification of TAK1 (*D*) and p65 (*E*) after LPS administration. *F*, representative images of p-TAK1 (*red*) and IBA (*green*) immunostaining in hippocampal CA1 (n = 3 in each group). Scale bar=50 μm. *G*, quantification of p-TAK1 immunoreactivity area in CA1. *H*, quantification of p-TAK1+IBA+ cells in IBA1+ microglia in CA1. *I*, representative images of p-TAK1 (*red*) and GFAP (*green*) immunostaining in hippocampal CA1 (n = 3 in each group). Scale bar=50 μm. One-way ANOVA followed by Tukey’s multiple-comparisons (*B*–*E*, *G*, *H*). ∗*p* < 0.05, ns, non-significant.

Recent studies emphasized the importance of TAK1 in mediating microglial responses during inflammation (20, 21). We also found that the levels of p-TAK1 (T187) and p-p65 (S536), but not total TAK1 or p65, increased after LPS administration (Fig. 2, *A*–*E*). The increases in phosphorylated TAK1 and p65 levels indicate a robust activation of the TAK1/NF-κB signaling pathway, which plays a critical role in the inflammatory response. Additionally, the p-TAK1 (T187) positive area, but not the percentage of p-TAK1+IBA+ cells, was elevated in the hippocampal CA1 (Fig. 2, *F*–*H*), CA3 (Fig. S2, *A*–C) and DG (Fig. S2, *D*–F) after LPS treatment in mice. Although TAK1 is expressed in astrocytes (35, 36), our findings showed that p-TAK1 (T187) was localized only in IBA1 positive cells (Fig. 2*F*; Fig. S2, *A* and *D*) and not in GFAP positive cells in CA1 (Fig. 2*I*), CA3, or DG regions (Fig. S2*G*). This suggests that TAK1 is exclusively activated in microglia and absent in astrocytes under our experimental conditions, highlighting its specific role in microglial activation during LPS-induced neuroinflammation.

### LPS increases neurotoxic astrocytes and limits newborn neurons generation

**Figure 3:**
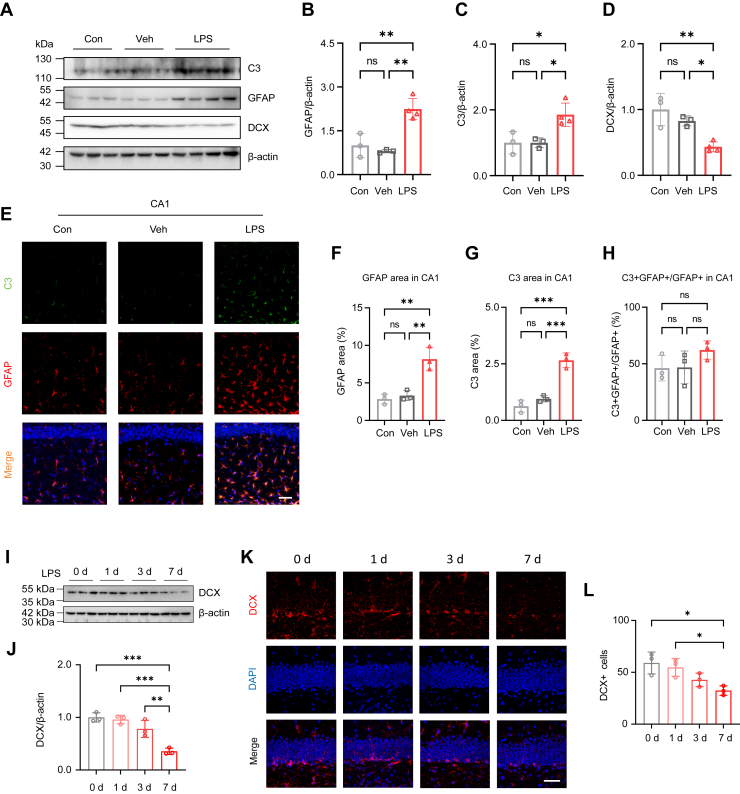
**LPS administration increases C3-positive astrocytes and decreases DCX-positive newborn neurons in the hippocampus.***A*, immunoblot analyses of GFAP, C3, and DCX in the hippocampus (n = 3 in Con and Veh, n = 4 in LPS). *B–D*, quantification of GFAP (*B*), C3 (*C*), and DCX (*D*) levels. *E*, representative images of C3 (*green*) and GFAP (*red*) immunostaining in hippocampal CA1 (n = 3 in each group). Scale bar=50 μm. *F*, quantification of GFAP immunoreactivity area in CA1. *G*, quantification of C3 immunoreactivity area in CA1. *H*, quantification of C3+GFAP+ cells in GFAP+ astrocytes in CA1. *I*, immunoblots of DCX in the hippocampus after LPS administration (n = 3 in each group). *J*, quantification of DCX levels. *K*, representative images of DCX positive cells in hippocampal DG (n = 3 in each group). Scale bar=50 μm. *L*, quantification of DCX-positive cells in DG. One-way ANOVA followed by Tukey’s multiple-comparisons test (*B*–*D*, *F*–*H*, *J*, *L*). ∗*p* < 0.05, ∗∗*p* < 0.01, ∗∗∗*p* < 0.001, ns, non-significant.

Activated microglia release pro-inflammatory cytokines and other mediators that stimulate neuroinflammation and drive astrocytes towards a neurotoxic phenotype known as A1-like astrocytes. This transformation involves an increase in neurotoxic factors, such as complement component 3 (C3), which exacerbates neuronal injury and contributes to synaptic dysfunction (16, 37, 38). After the i.p. administration of LPS for 7 days, immunoblots revealed a significant increase in both GFAP and C3 levels (Fig. 3, *A*–*C*) and a decrease in DCX levels (Fig. 3, *A* and *D*). C3-positive signals co-localized with GFAP-positive cells in the hippocampal CA1 (Fig. 3*E*), CA3 (Fig. S3*A*), and DG (Fig. S3*E*) regions. The positive area of GFAP and C3, but not the percentage of C3+GFAP+ cells, was also upregulated in the hippocampus (Fig. 3, *F*–*H*; Fig. S3, *B*–*D*, *F*–*H*). Thus, these results prove that LPS leads to an increase in C3 positive neurotoxic astrocytes in the hippocampus.

The protein levels of NeuN, SYN, SYP, and PSD95 remained unchanged following 7 days of LPS administration (Fig. S3, *I*–*M*), but LPS administration reduced the levels of DCX (Fig. 3, *A*, *D*, *I* and *J*). The number of DCX-positive cells was also significantly reduced in the hippocampal DG (Fig. 3, *K* and *L*). This reduction in DCX-positive cells suggests a possible disruption in the neurogenic process, which may significantly impair cognitive functions.

### TAK1 activation induces neurotoxic astrocytes and damages hippocampal neurons *in vitro*

**Figure 4:**
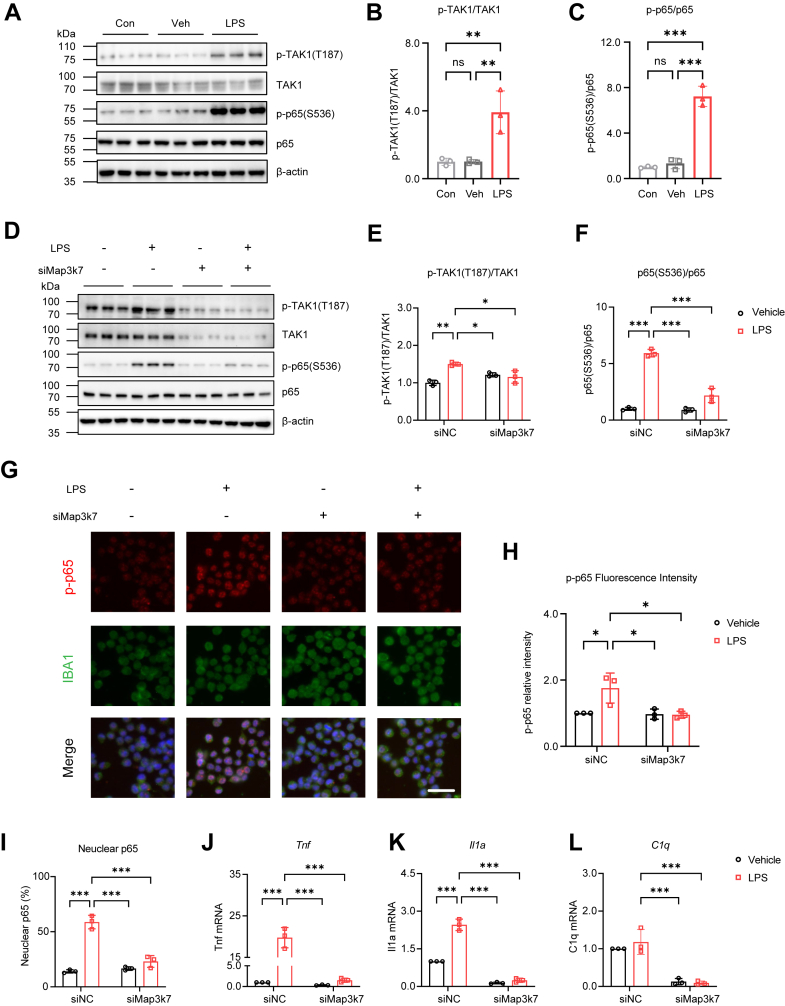
**TAK1 knockdown inhibits inflammatory responses in LPS-stimulated BV2 cells.***A*, immunoblot analyses of p-TAK1 (T187) and p-p65 (S536) in the untreated control group (Con), vehicle group (Veh), and LPS group (LPS) (n = 3 in each group). *B*, quantification of p-TAK1 (T187) in LPS-treated BV2 cells. *C*, quantification of p-p65 (S536) in LPS-treated BV2 cells. *D*, immunoblot analyses of p-TAK1 (T187) and p-p65 (S536) in siRNA-treated BV2 cells (n = 3 in each group). *E*, quantification of p-TAK1 (T187) in siRNA-treated BV2 cells. *F*, quantification of p-p65 (S536) in siRNA-treated BV2 cells. *G*, the relative fluorescence intensity of p-p65 (S536) (*red*) was investigated in IBA1 positive BV2 cells (*green*) (n = 3 in each group). Scale bar=50 μm. *H*, quantification of relative fluorescence intensity of p-p65 in BV2 cells. *I*, quantification of nuclear p65 proportion in siRNA-treated BV2 cells. *J–L*, quantification of *Tnf* (*J*), *Il1a* (*K*), and *C1q* (*L*) mRNA in siRNA-treated BV2 cells. One-way ANOVA followed by Tukey’s multiple-comparisons test (*B* and *C*). Two-way ANOVA followed by Tukey's multiple-comparisons test (*E*, *F*, *H*–*L*). ∗*p* < 0.05, ∗∗*p* < 0.01, ∗∗∗*p* < 0.001, ns, non-significant.

To further investigate the role of LPS-activated TAK1 in microglia and to determine whether these activated microglia induce neurotoxic astrocytes and damage hippocampal neurons, we first measured p-TAK1 levels following LPS treatment at different time points *in vitro*. Protein levels of p-TAK1 (T187) and p-p65 (S536), but not total TAK1 and p65, were elevated in LPS-treated BV2 cells at 15 min (Fig. S4, *A*–*E*), and peaked at 30 min compared to cells in the untreated control (Con) or vehicle (Veh) groups (Fig. 4, *A*–*C*; Fig. S4, *F* and *G*).

After verifying the knockdown efficiency of *Map3k7* siRNA (siMap3k7) in BV2 cells by detecting the *Map3k7* mRNA expression (Fig. S4*H*), we employed *Map3k7* RNA interference (RNAi) to reduce TAK1 expression in BV2 cells and examined whether TAK1 knockdown affects LPS-activated NF-κB/p65 (Fig. S4*I*). After 30 min of LPS exposure, the levels of p-TAK1 (T187) and p-p65 (S536) significantly decreased in siMap3k7-treated cells compared to siNC-treated cells (Fig. 4, *D*–*F*). Moreover, the levels of TAK1 were significantly reduced in siMap3k7-treated cells, but the levels of total p65 did not change (Fig. 4*D*; Fig. S4, *J* and *K*). Then, the relative fluorescence intensity of p-p65 (S536) was examined in IBA1 positive BV2 cells (Fig. 4*G*). In LPS-stimulated BV2 cells, the fluorescence intensity of p-p65 increased, which was largely prevented by siMap3k7 (Fig. 4*H*). Similarly, the increased nuclear p65 in LPS-stimulated BV2 cells was diminished by TAK1 knockdown (Fig. S4*L*; Fig. 4*I*). After 3 h treatment with LPS, the mRNA expression of *Tnf* and *Il1a* was elevated in siNC-treated cells, while it decreased in siMap3k7-treated cells (Fig. 4, *J* and *K*). *C1q* mRNA levels did not rise after LPS stimulation, but they still decreased in siMap3k7-treated cells (Fig. 4*L*). Overall, these results demonstrate that TAK1 modulates the inflammatory response in LPS-stimulated BV2 cells.

**Figure 5:**
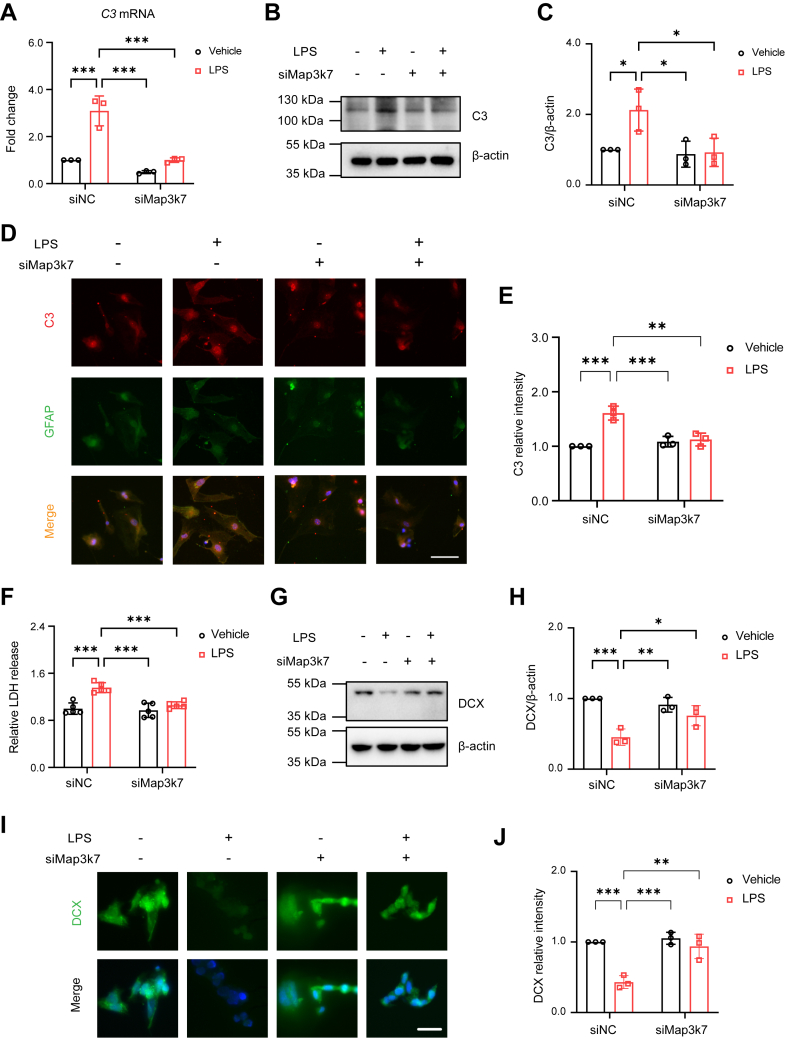
**Effects of microglia conditioned medium (MCM) on mouse astrocytes and HT22 cells.***A*, quantification of *C3* mRNA in MCM-treated astrocytes (n = 3 in each group). *B*, immunoblots of C3 in MCM-treated astrocytes (n = 3 in each group). *C*, quantification of C3 level in MCM-treated astrocytes. *D*, the relative fluorescence intensity of C3 (*red*) was investigated in GFAP positive astrocytes (*green*) (n = 3 in each group). Scale bar=100 μm. *E*, quantification of C3 relative intensity in MCM-treated astrocytes. *F*, quantification of LDH release level in MCM-treated HT22 cells (n = 5 in each group). *G*, immunoblots of DCX in MCM-treated HT22 cells (n = 3 in each group). *H*, quantification of DCX level. *I*, the relative fluorescence intensity of DCX was investigated in HT22 cells (n = 3 in each group). Scale bar=50 μm. *J*, quantification of DCX relative fluorescence intensity. Two-way ANOVA followed by Tukey's multiple-comparisons test (*A*, *C*, *E*, *F*, *H*, *J*). ∗*p* < 0.05, ∗∗*p* < 0.01, ∗∗∗*p* < 0.001.

Next, we assessed whether TAK1 reduction diminished neurotoxic astrocytes *in vitro.* The microglia conditioned medium (MCM) from LPS-simulated BV2 cells was prepared as shown in Figure S4*M*. After treatment with MCM derived from LPS-stimulated BV2 cells for 24 h, we found that both C3 mRNA and protein were significantly elevated in cultured mouse astrocytes, while they were reduced in MCM from siMap3k7-treated BV2 cells (Fig. 5, *A*–*C*). Moreover, we assessed the relative fluorescence intensity of C3 in cultured GFAP positive astrocytes. The results showed that C3 fluorescence intensity increased in the MCM from LPS-treated BV2 cells but decreased in MCM from siMap3k7-treated BV2 cells (Fig. 5, *D* and *E*).

The cytotoxicity effects of MCM on HT22 cells were then evaluated. After treating HT22 cells with MCM derived from LPS-stimulated BV2 cells, we observed an increase in released LDH levels, whereas the LDH levels remained unchanged in HT22 cells treated with MCM from siMap3k7-treated BV2 cells (Fig. 5*F*). Immunoblots revealed that DCX levels were lower in MCM from LPS-treated BV2 cells, but not in MCM from siMap3k7-treated BV2 cells (Fig. 5, *G* and *H*). Similarly, the relative fluorescence intensity of DCX decreased in MCM from LPS-treated BV2 cells, but it remained unchanged in MCM from siMap3k7-treated BV2 cells (Fig. 5, *I* and *J*). In summary, these findings demonstrate that LPS-induced TAK1 activation in BV2 cells not only enhances the neurotoxic phenotype of astrocytes but also contributes to the cytotoxic effects on HT22 cells *in vitro*.

### TAK1 reduction improves animal behavioral performance and suppresses microglial activation *in vivo*

**Figure 6:**
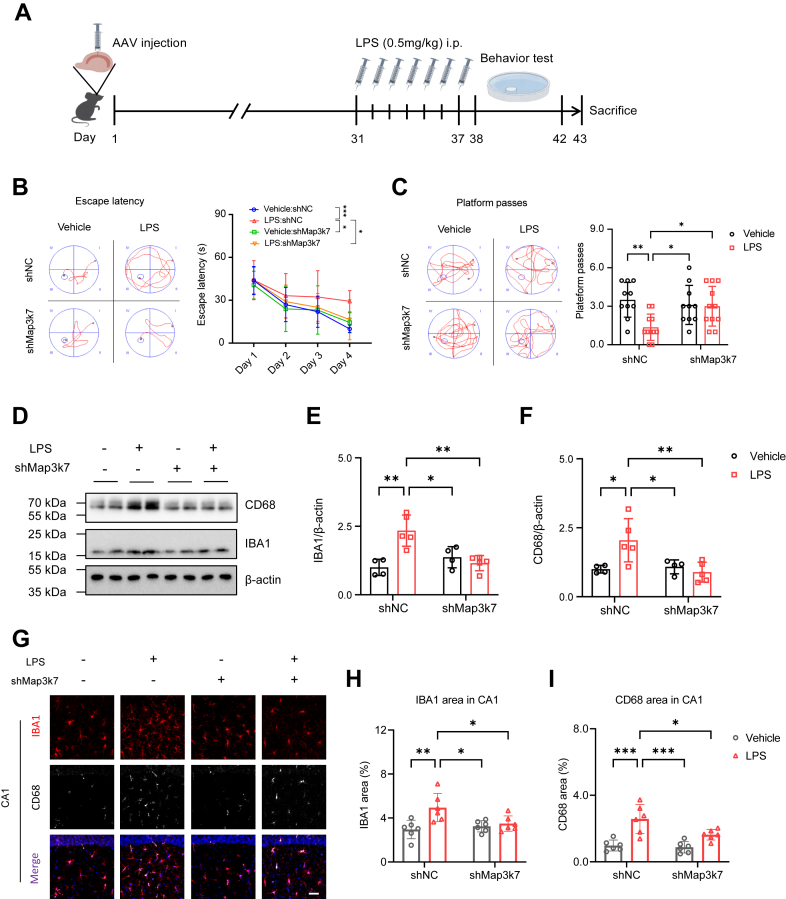
**TAK1 reduction improves behavioral deficits and alleviates microglial activation.***A*, a schematic diagram of the animal experimental procedure. *B* and *C*, quantification of escape latency in acquisition trial (*B*) and platform passes in probe trial (*C*) (n = 10 in Vehicle:shNC and Vehicle:shMap3k7, n = 11 in LPS:shNC and LPS:shMap3k7). *D*, immunoblots of IBA1 and CD68 in the hippocampus (n = 4 in Vehicle:shNC and Vehicle:shMap3k7, n = 5 in LPS:shNC and LPS:shMap3k7). *E* and *F*, quantification of IBA1 (*E*) and CD68 (*F*) levels. *G*, representative images of IBA (*red*) and CD68 (*white*) immunostaining in hippocampal CA1 (n = 6 in each group). Scale bar=50 μm. *H* and *I*, quantification of IBA1 (*H*) and CD68 (*I*) immunoreactivity area in CA1. Kruskal-Wallis test followed by Dunn’s multiple-comparisons test (*B*). Two-way ANOVA followed by Tukey's multiple-comparisons test (*C*, *E*, *F*, *H*, *I*). ∗*p* < 0.05, ∗∗*p* < 0.01, ∗∗∗*p* < 0.001.

Next, we examined whether targeting TAK1 could be a potential therapeutic strategy to reduce neuroinflammation and improve behavioral performance in animals. Targeting microglia with recombinant AAV (rAAV) is a prevalent strategy for studying microglial pathophysiology and therapies (39, 40, 41), and AAV9 driven by the IBA1 promoter can be successfully transduced into microglia (40, 42, 43, 44). In this study, AAV was injected into the mouse hippocampus as illustrated in Figure 6*A*. We observed that zsGreen signals co-localized with IBA1-positive microglia in the hippocampal CA1, CA3, and DG regions 30 days after AAV injection (Fig. S5, *A* and *B*). The AAV-shMap3k7 reduced *Map3k7* mRNA expression *in vivo* (Fig. S5*C*). Following 7 days of LPS administration, the MWM test was conducted to assess animal behavior. LPS administration led to an increase in escape latency, which was reduced in animals receiving AAV-shMap3k7 injection (Fig. 6*B*). Additionally, the number of platform passes decreased in LPS-administered mice but increased in those injected with AAV-shMap3k7 (Fig. 6*C*). However, swimming speed, as well as time and distance in the target zone, remained consistent across groups (Fig. S5, *D*–F).

Immunoblots confirmed that AAV-shMap3k7 reduced TAK1 protein levels (Fig. S5, *G* and *H*). Moreover, elevated levels of IBA1 and CD68 (Fig. 6, *D*–*F*), as well as COX2 and iNOS (Fig. S5, *G*, *I*, and *J*), induced by LPS were significantly decreased in the hippocampus of AAV-shMap3k7-treated mice. In the hippocampal CA1 region, IBA1 and CD68 positive areas (Fig. 6, *G*–*I*) were also reduced in mice with AAV-shMap3k7 injection compared to those treated with LPS. Similar results were found in CA3 (Fig. S6, *A*–C) and DG (Fig. S6, *D*–F). These findings indicate that TAK1 knockdown alleviates LPS-induced behavioral impairments and suppresses microglial activation *in vivo*.

### TAK1 reduction limits neurotoxic astrocytes and restores the loss of newborn neurons

**Figure 7:**
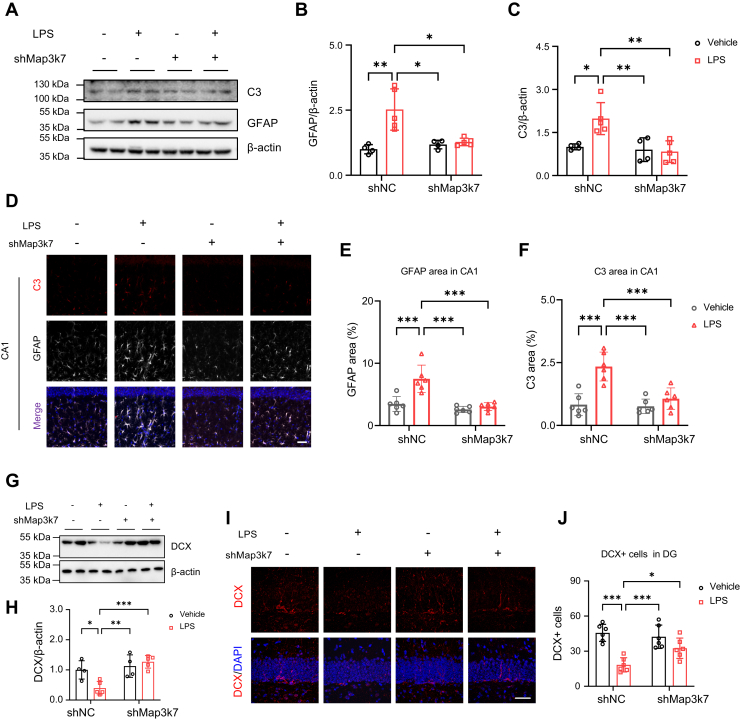
**TAK1 reduction limits C3 positive astrocytes and rescues DCX positive newborn neurons.***A*, immunoblots of GFAP and C3 in hippocampus (n = 4 in Vehicle:shNC and Vehicle:shMap3k7, n = 5 in LPS:shNC and LPS:shMap3k7). *B* and *C*, quantification of GFAP (*B*) and C3 (*C*) levels. *D*, representative images of C3 (*red*) and GFAP (*white*) immunostaining in hippocampal CA1 (n = 6 in each group). Scale bar=50 μm. *E* and *F*, quantification of GFAP (*E*) and C3 (*F*) immunoreactivity area in CA1. *G*, immunoblots of DCX in hippocampus (n = 4 in Vehicle:shNC and Vehicle:shMap3k7, n = 5 in LPS:shNC and LPS:shMap3k7). *H*, quantification of DCX levels. *I*, immunofluorescence of DCX positive cells in DG. Scale bar=50 μm. *J*, Quantification of DCX positive cells in DG. Two-way ANOVA followed by Tukey's multiple-comparisons test (*B*, *C*, *E*, *F*, *H*, *J*). ∗*p* < 0.05, ∗∗*p* < 0.01, ∗∗∗*p* < 0.001.

Finally, we investigated whether reducing TAK1 limited neurotoxic astrocytes and mitigated the loss of newborn neurons. TAK1 reduction *via* AAV-shMap3k7 administration decreased the LPS-induced GFAP and C3 levels (Fig. 7, *A*–*C*). Both GFAP and C3 positive staining areas in CA1 (Fig. 7, *D*–*F*), CA3 (Fig. S7, *A*–C), and DG (Fig. S7, *D*–*F*) were significantly reduced with AAV-shMap3k7 injection. Additionally, immunoblots further demonstrated that reducing TAK1 restored DCX levels (Fig. 7, *G* and *H*), thereby protecting against the loss of newborn neurons in the DG (Fig. 7, *I* and *J*). Overall, these findings indicate that the reduction of microglial TAK1 not only limits the activation of neurotoxic astrocytes but also mitigates neuronal loss.

## Discussion

Neuroinflammation, characterized by the activation of glial cells, contributes significantly to neuronal injury and the progression of cognitive decline (45, 46). Microglia respond to pathological stimuli by releasing pro-inflammatory cytokines, which can exacerbate neurotoxicity, leading to neuronal death and cognitive impairment (2, 8). Systemic LPS administration induces significant neuroinflammation in the hippocampus, characterized by microglial activation and elevated levels of pro-inflammatory cytokines (29, 47). Evidence suggests that microglia also have neuroprotective roles, as they clear toxic aggregates such as tau and α-synuclein (48). Therefore, understanding these effects is crucial for developing therapeutic strategies aimed at reducing hippocampal damage linked to systemic inflammation.

In this study, we investigated the role of TAK1 in LPS-induced microglial activation and its effects on astrocytes. Our findings revealed that LPS significantly activated microglia in the hippocampus, as evidenced by increased levels of IBA1 and CD68. This activation was accompanied by elevated phosphorylation of TAK1 and downstream effector NF-κB/p65, indicating a robust inflammatory response. Additionally, the MCM from activated BV2 cells showed cytotoxic effects on HT22 neuronal cells, emphasizing the harmful impact of microglial activation on neuronal health. Overall, these results indicate that systemic LPS leads to TAK1 activation, making TAK1 a potential therapeutic target for modulating neuroinflammation.

The interaction between microglia and astrocytes plays a crucial role in neuroinflammation. TNFα and IL-1α derived from microglia are known to induce A1-like astrocytes, a neurotoxic phenotype that exacerbates neuronal degeneration by releasing C3 (16, 49, 50). The complement system, particularly the protein C3, plays a crucial role in mediating the effects of activated astrocytes on neuronal health. Astrocyte-derived C3 interacts with neuronal C3 receptors, leading to synaptic damage and impairments in neuronal function (37, 38, 51, 52). Astrocyte-derived C3 also stimulates microglia through C3 receptors, promoting them to secrete complement C1q and pro-inflammatory cytokines, which further contribute to neuronal degeneration. This astrocyte-microglia crosstalk amplifies neuronal damage and promotes neurodegeneration (53, 54).

TAK1 is a key component in several signaling pathways related to inflammation and neurodegeneration (25, 26, 55). Our findings show that LPS-stimulated BV2 cells increased the mRNA expression of *Tnf* and *Il1a*, alongside significantly elevated C3 levels in astrocytes. However, the absence of TAK1 activation reduced LPS-induced microglial activation and decreased C3 levels in astrocytes. This is consistent with recent studies that emphasize the role of inflammatory factors in promoting a neurotoxic astrocyte phenotype (56, 57). Our results suggest that TAK1 activation not only amplifies the inflammatory response but also promotes microglia-astrocyte cross-talk, driving the production of neurotoxic factors that may contribute to neuronal loss. Moreover, the interaction between microglia and astrocytes, especially through the modulation of TNFα, IL1-α, and C3, plays a crucial role in the development of neuroinflammatory diseases. This further emphasizes the potential function of TAK1 as a therapeutic target for mitigating neuroinflammation.

LPS induces neuroinflammation, especially in the hippocampus, which is crucial for learning and memory processes (58). In this study, the unchanged levels of established neuronal markers such as NeuN, SYN, SYP, and PSD95 indicated that the integrity and function of mature neurons remain largely unaffected by short-term low-dose LPS exposure. Previous studies indicate that inflammation can lead to a hostile neurogenic niche in the brain, affecting the survival and maturation of newborn neurons and aggravating cognitive dysfunction (58, 59, 60, 61). In this study, the observed LPS-induced reduction of DCX positive cells in the hippocampal DG suggests a potential impairment in neurogenesis. However, AAV-mediated TAK1 reduction prevented the loss of newborn neurons. Additionally, in the behavioral tests, mice with AAV-driven TAK1 inhibition exhibited shorter escape latency and increased entries into the target zone, which indicates enhanced spatial learning and memory. These findings suggest that TAK1 suppression restores the loss of newborn neurons and improves cognitive functions directly. Our results indicate that TAK1 promotes glial activation, fostering a detrimental hippocampal environment for neurogenesis.

Overall, this study highlights the role of TAK1 in LPS-induced neuroinflammation, which implies a molecular mechanism that links microglial activation to neuronal loss. Our findings reveal how microglia and astrocytic responses interact, identifying TAK1 as a potential target for mitigating neuroinflammation. The interaction between TAK1 activation, microglial responses, and neurotoxic astrocyte transition deepens our understanding of the cellular dynamics driving LPS-induced neuroinflammation.

## Experimental procedures

### Animals

Male C57BL/6 mice (age 6–8 weeks, 20–25 g) were purchased and bred at the Animal Laboratory of Nantong University. All mice were raised in a 12-h light/dark cycle with free access to water and food. All animal experiments were performed after randomization. LPS (Servicebio) was resuspended in sterile saline at a concentration of 0.1 mg/ml, mice were administered with LPS (0.5 mg/kg per day) *via* i.p. for 1, 3, or 7 days. Mice in the vehicle group received sterile saline at the same volume. Subsequently, the brain tissues were harvested for further analysis. All procedures were conducted in accordance with the ethical guidelines approved by the Institutional Ethical Committee of Nantong University (Approved number: S202110224-037).

### Cell culture

Murine microglial cell line BV2 (Pricella) and hippocampal neuronal cell line HT22 (Cellverse) were cultured in Dulbecco’s modified Eagle’s medium (DMEM) (Servicebio) supplemented with 10% fetal bovine serum (FBS) (Beyotime) and 1% penicillin-streptomycin (Beyotime). To eliminate any remaining FBS interference, BV2 cells were cultured in fresh DMEM for 1 hour before being treated with 100 ng/ml LPS (Servicebio). Cells in the vehicle group received PBS at the same volume.

Primary mouse astrocytes were prepared as described in the protocol with some modifications (62). Briefly, primary astrocytes were isolated from the hippocampi of C57BL/6 mouse pups at postnatal day 3. After the removal of the meninges, all tissues were washed three times in PBS. Then, the tissues were transferred to 0.25% Trypsin-EDTA (Beyotime) and gently agitated for 5 min. Tryptic-digested samples were passed through a 70-μm nylon mesh and centrifuged at 1200 rpm for 5 min. Cell precipitates were resuspended in DMEM with 10% FBS and cultured in T75 flasks coated with Poly-L-lysine (PLL) (Bomeibio) for 7 days. Non-astrocytic cell populations were removed by shaking at 160 rpm for 60 min, and the culture medium containing the removed cells was discarded. The purified astrocytes were identified using GFAP staining.

### RNA interference (RNAi)

Negative control siRNA (siNC) and *Map3k7* siRNA (siMap3k7) for RNAi in BV2 cells were purchased from Ribobio (Guangzhou, China). The target sequence for siMap3k7 is 5′-ATG GAC ATT GCT TCT ACA AAT-3′. 2 × 10^4^ or 1.5 × 10^5^ cells were seeded into either 24-well plates or 6-well plates in DMEM with 10% FBS for 24 h. According to the manufacturer’s instructions, we used Lipo8000 Transfection Reagent (Beyotime) to transfect 50 nM siRNA into BV2 cells for 4 h. Subsequently, we replaced the medium with fresh DMEM containing 10% FBS. Forty-eight hours later, all cells were treated with either LPS or PBS and then harvested them for further assays.

### Microglia conditioned medium (MCM) preparation

To prepare MCM, 1.5 × 10^5^ BV2 cells were placed on a 6-well plate with 2 ml of DMEM containing 10% FBS for 24 h. Next, all BV2 cells were treated with LPS (100 ng/ml) in DMEM for 3 h. After treatment, the cells were washed three times with PBS to remove any residual LPS. The BV2 cells were then replaced with fresh DMEM medium for another 24 h. Finally, MCM was collected and centrifuged at 1000 rpm for 15 min to remove cellular debris.

### Stereotaxic injection

AAV2/9-IBA1-shMap3k7-zsGreen (AAV-shMap3k7) and negative control AAV2/9-IBA1-shRNA-zsGreen (AAV-shNC) were obtained from Hanbio at a final concentration of 1 × 10^13^ vg/ml. Animals were placed in a stereotaxic frame and received a total volume of 0.5 μl of viral vectors per injection site at a rate of 0.1 μl/min using a micro-injector. The stereotaxic coordinates used were as follows: anteroposterior (AP) −2.0 mm; mediolateral (ML) +1.5 mm; dorsoventral (DV) −1.8 mm from bregma. Thirty days post-injection, further experiments were performed.

### Morris water maze (MWM) test

The MWM was used to analyze spatial learning and memory performance. Three days before the experiment, all mice were handled for at least 5 min per day. The testing room was kept quiet. To adapt to the environment, all mice were placed in the room at least 2 h before MWM tests. The water maze consisted of a pool (120 cm in diameter) containing opaque water (20 °C ± 1  deg) and an invisible platform (10 cm in diameter) 1.5 cm below the surface. The pool was artificially divided into four virtual quadrants (N, S, E, W) facing the wall of the tank. During the training period, the mice were allowed to freely swim for 60 s to find the platform. The mice that found the platform within 60 s were allowed to stay for 30 s. If a mouse failed to find the platform, it was guided to the platform and maintained there for 30 s. The probe trial was performed 24 h after the last training trial in order to test the memory retention of the mice. During the trial, the platform was removed, and the mice were allowed to freely explore for 60 s. A camera was mounted above the maze to record the swimming traces in the test.

### Real-time quantitative PCR (RT-qPCR)

Total RNA extraction and cDNA preparation were performed from cells or tissues in accordance with the manufacturer’s instructions for the RNAeasy Animal RNA Isolation Kit (Beyotime) and the BeyoRT II First Strand cDNA Synthesis Kit (Beyotime). RT-qPCR was then directly monitored using ABI Stepone plus system (Applied Biosystem) with BeyoFast SYBR Green qPCR Mix (2×, High ROX) (Beyotime). The primer sequences of reference gene *18s* rRNA and target genes *Tnf*, *Il1a*, *C1q*, and *C3* were described in previous reports (17, 63). The primers used for *Map3k7* were as follows: 5′-TTACTACACTGCTGCTCATGCC-3′/5′-AACCAGCAGCAAGTTTGGAGG-3′. All oligonucleotides were obtained from Sangon Biotech. The relative mRNA expression of target genes in each sample were calculated and analyzed using the 2^−ΔΔ CT^ method (64).

### Protein extraction and Western blot (WB) analysis

Total protein from hippocampal tissues and cultured cells was homogenized in RIPA Lysis Buffer (Beyotime) containing CLAP protease inhibitors mix (Bomeibio) and PMSF (Bomeibio). WB was performed as previously described (63). Briefly, protein lysates were separated by SDS-PAGE and transferred to PVDF membranes. The PVDF membranes were blocked with 5% skim milk in 1×TBST for 1 h at room temperature and incubated overnight with primary antibodies at 4 °C. Next, the membranes were incubated with appropriate HRP-conjugated secondary antibodies for 1 h. Protein bands were visualized using the Chemi-Doc XRS imaging system (Bio-Rad). Semi-quantitative densitometry analyses are performed by ImageLab software (Bio-Rad). The antibodies used are listed in Table S1.

### Immunofluorescence (IF) staining

Animals were perfused *via* the heart with 0.9% normal saline (NS) and fixed with 4% paraformaldehyde (PFA). Excised brains were post-fixed overnight in 4% PFA and dehydrated in 20% and 30% sucrose for 24 h. Hippocampal tissue was sliced into 30 μm-thick coronal frozen sections and stored in a storage solution at 4 °C. Sections from the storage solution were incubated in 0.5× Antigen Retrieval Solution for Frozen Sections (Beyotime) for 3 min. All cultured cells were seeded in 24-well plates and fixed in 2% PFA at room temperature for 5 min. For IF, all sections or cells were washed in PBS and incubated in a blocking solution (5% bovine serum albumin, 0.1% Triton X-100 in PBS) for 1 h at room temperature. DAPI was used to stain the cell nuclei. Sections from all groups were stained and analyzed with the same microscope settings. Fluorescent images from different hippocampal regions (CA1, CA3, DG) were acquired using a Zeiss Laser Microscope (Zeiss) or an Olympus Laser Confocal Microscope (Olympus) with a 40× or 60× objective. Area fractions were measured on a 0.1 mm^2^ area using FV10-ASW software (Olympus), or positive staining was measured on a 0.09 mm^2^ area using Zen3.2 software (Zeiss). Color channels were split, and a fixed threshold was automatically determined using the Moments algorithm in Fiji software (NIH), and the threshold area over the total image area was further calculated. Both image and cell quantification were performed in a blind manner to ensure objectivity. The antibodies used are listed in Table S2.

### LDH release assay

The cytotoxicity of HT22 cells was examined using the LDH Cytotoxicity Assay Kit (Beyotime). In brief, 5 × 10^3^ HT22 cells were cultured in 96-well plates in DMEM with 10% FBS for 24 h. Next, all HT22 cells were washed with PBS three times to eliminate any remaining FBS. After MCM incubation for 24 h, HT22 cells were cultured with 120 μl fresh DMEM for another 24 h. Next, 100 μl of growth medium collected from HT22 cells was mixed with 50 μl LDH detection working solution and shaken for 30 min at room temperature. The absorbance was measured at 490 nm by a Synergy 2 enzyme mark instrument (BioTek) to determine cell cytotoxicity.

### Statistical analysis

Data are presented as the mean ± standard deviation (SD). The normality of distribution was tested using the Shapiro-Wilk test, and the equality of variance was confirmed using the F-test. The parametric tests used were two-tailed unpaired Student’s *t* test or unpaired *t* test with Welch’s correction. One-way analysis of variance (ANOVA) followed by Dunnett’s or Tukey’s multiple comparison tests, as well as the Brown-Forsythe and Welch ANOVA test. Two-way ANOVA followed by Tukey’s multiple comparison tests. The non-parametric tests used were the Kruskal–Wallis test followed by Dunn’s multiple comparison test. The statistical analyses were performed using GraphPad Prism 9.5 (GraphPad Software). *p*-values are represented as ∗*p* < 0.05, ∗∗*p* < 0.01, ∗∗∗*p* < 0.001.

## Data availability

The data used and analyzed during the current study are available from the corresponding author upon reasonable request.

## Supporting information

This contains supporting information.

## Conflict of interest

The authors declare that they have no conflicts of interest with the contents of this article.
