# Supplementary material for: Microglial TAK1 promotes neurotoxic astrocytes and cognitive impairment in LPS-induced hippocampal neuroinflammation
Source: J Biol Chem. 2025 May 9;301(6):110225. doi: 10.1016/j.jbc.2025.110225 (PMC12179612; doi:10.1016/j.jbc.2025.110225)
Supplement: Supplementary Tables [file mmc1.docx]

**Table S1. List of antibodies used in Western blot.**

| **Antibodies** | **Source** | **Identifier** |
| --- | --- | --- |
| Goat anti-IBA1 1:500 | Abcam | Ab5076 |
| Rabbit anti-CD68 1:2000 | Abclonal | A6554 |
| Rat anti-GFAP 1:3000 | Abcam | Ab279291 |
| Rabbit anti-C3 1:1000 | ThermoFisher | PA5-21349 |
| Rabbit anti-DCX 1:1000 | Beyotime | AF6675 |
| Rabbit anti-NeuN 1:1000 | Abcam | Ab177487 |
| Rabbit anti-SYN 1:1000 | Zenbio | 340966 |
| Rabbit anti-SYP 1:2000 | Beyotime | AF8091 |
| Rabbit anti-COX2 1:1000 | Beyotime | AF1924 |
| Rabbit anti-iNOS 1:1000 | Beyotime | AF7281 |
| Rabbit anti-PSD95 1:1000 | Abclonal | A7889 |
| Rabbit anti-p-TAK1 (T187) 1:1000 | Abclonal | AP1222 |
| Rabbit anti-p-NF-κB/p65 (S536) 1:500 (Tissue) | Beyotime | AF5881 |
| Rabbit anti-p-NF-κB/p65 (S536) 1:1000 (Cell) | CST | 3033 |
| Rabbit anti-TAK1 1:1000 | Proteintech Group | 12330-2-AP |
| Rabbit anti-NF-κB/p65 1:1000 | CST | 8242 |
| Mouse-anti-β-actin 1:2500 | Abclonal | AC004 |
| HRP-conjugated Goat Anti-Rabbit IgG 1:5000 | Sangon | D110058 |
| HRP-conjugated Goat Anti-Mouse IgG 1:5000 | Beyotime | A0350 |
| HRP-conjugated Goat Anti-Rat IgG 1:5000 | Beyotime | A0356 |

**Table S2. List of antibodies used in Immunofluorescence.**

| **Antibodies** | **Source** | **Identifier** |
| --- | --- | --- |
| Goat anti-C3d 1:200 | R&D | AF2655 |
| Rabbit anti-CD68 1:200 | Servicebio | GB113109 |
| Rabbit anti-DCX 1:100 | Abcam | Ab18723 |
| Mouse anti-GFAP 1:500 | Beyotime | AF0156 |
| Rabbit anti-GFAP 1:200 | Sangon | D262817 |
| Goat anti-IBA1 1:600 | Abcam | Ab5076 |
| Rabbit anti-p-TAK1(T187) 1:100 | CST | 4536S |
| Mouse anti-NF-κB/p65 1:200 | Abclonal | A10609 |
| Rabbit anti-p-NF-κB/p65(S536) 1:200 | Beyotime | AF5881 |
| Cy3-conjugated Donkey Anti-Goat IgG 1:1000 | Servicebio | GB21404 |
| Alexa Fluor 488-conjugated Donkey Anti-Goat IgG 1:1000 | Servicebio | GB25404 |
| FITC-conjugated Donkey Anti-Rabbit IgG 1:1000 | Servicebio | GB22403 |
| Alexa Fluor 555-conjugated Donkey Anti-Rabbit IgG 1:1000 | Beyotime | A0453 |
| FITC-conjugated Donkey Anti-Mouse IgG 1:1000 | Servicebio | GB22401 |
| Alexa Fluor 555-conjugated Donkey Anti-Mouse IgG 1:1000 | Beyotime | A0460 |
| CoraLite647-conjugated Donkey Anti-Rabbit IgG 1:1000 | Proteintech Group | SA00014-7 |
| DAPI | Servicebio | G1012 |
