# Supplementary material for: Microglial TAK1 promotes neurotoxic astrocytes and cognitive impairment in LPS-induced hippocampal neuroinflammation
Source: J Biol Chem. 2025 May 9;301(6):110225. doi: 10.1016/j.jbc.2025.110225 (PMC12179612; doi:10.1016/j.jbc.2025.110225)
Supplement: Supplementary Figures [file mmc2.docx]

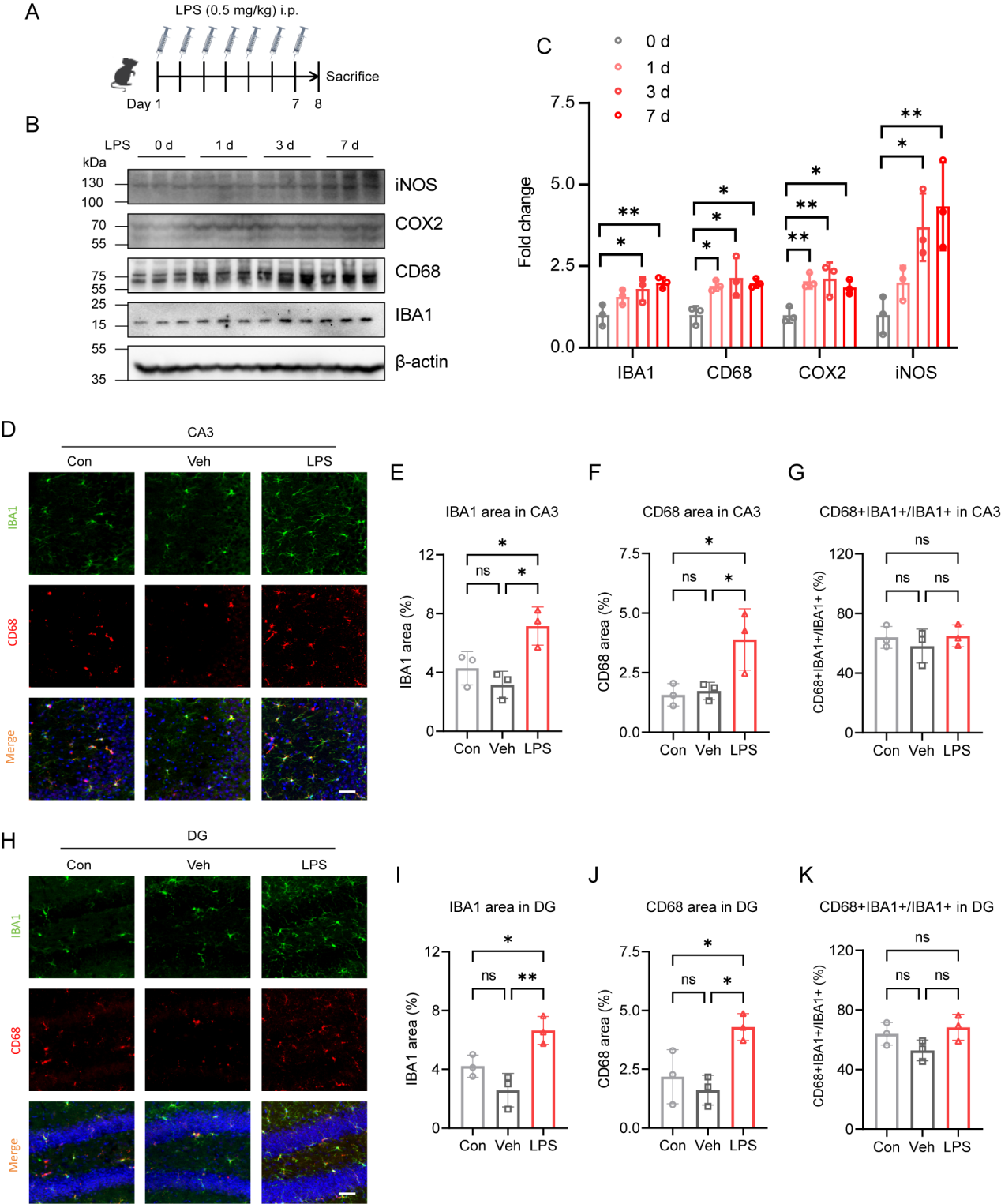


**Fig.S1 LPS administration activates microglia in the hippocampus. Related to Figure 1.**

1. A schematic overview of LPS i.p. administration.
2. Immunoblot analyses of IBA1, CD68, COX2, and iNOS in the hippocampus at different time points (n=3 in each group).
3. Quantification of IBA1, CD68, COX2, and iNOS levels.
4. Representative images of IBA (green) and CD68 (red) immunostaining in hippocampal CA3 (n=3 in each group). Scale bar=50 μm.
5. Quantification of IBA1 immunoreactivity area in CA3.
6. Quantification of CD68 immunoreactivity area in CA3.
7. Quantification of CD68+IBA+ cells in IBA1+ microglia in CA3.
8. Representative images of IBA (green) and CD68 (red) immunostaining in hippocampal DG (n=3 in each group). Scale bar=50 μm.
9. Quantification of IBA1 immunoreactivity area in DG.
10. Quantification of CD68 immunoreactivity area in DG
11. Quantification of CD68+IBA+ cells in IBA1+ microglia in DG.

One-way ANOVA followed by Dunnett’s multiple-comparisons test (C). One-way ANOVA followed by Tukey’s multiple-comparisons test (E-G, I-K). **p*<0.05, ***p*<0.01, ns: non-significant.


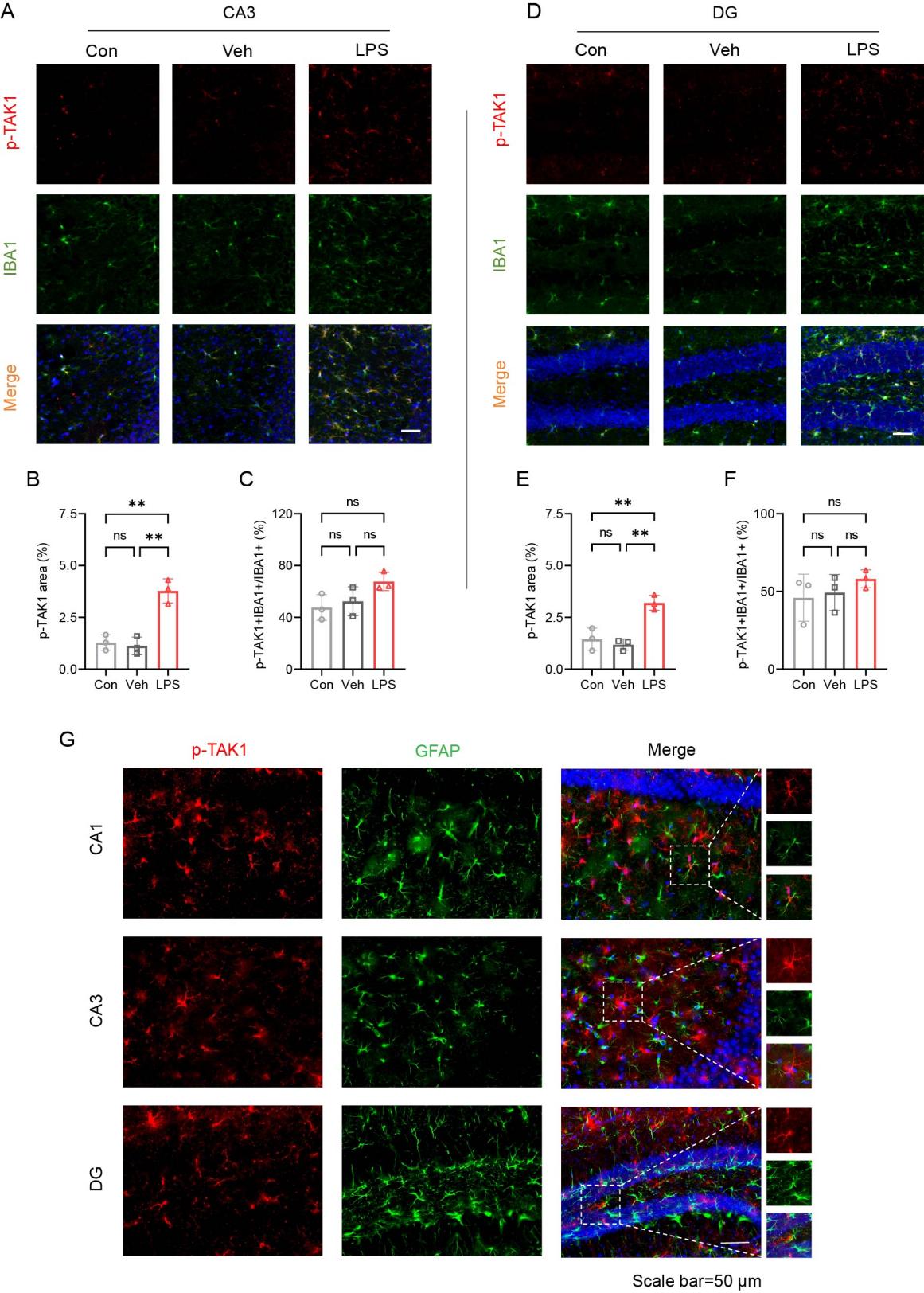


**Fig.S2 LPS administration induces microglial p-TAK1 activation in the hippocampus. Related to Figure 2.**

1. Representative images of p-TAK1 (red) and IBA1 (green) immunostaining in hippocampal CA3 (n=3 in each group). Scale bar=50 μm.
2. Quantification of p-TAK1 immunoreactivity area in CA3.
3. Quantification of p-TAK1+IBA+ cells in IBA1+ microglia in CA3.
4. Representative images of p-TAK1 (red) and IBA1 (green) immunostaining in hippocampal DG (n=3 in each group). Scale bar=50 μm.
5. Quantification of p-TAK1 immunoreactivity area in DG.
6. Quantification of p-TAK1+IBA+ cells in IBA1+ microglia in DG.
7. Representative images of p-TAK1 (red) and GFAP (green) immunostaining in hippocampal DG after LPS administration (n=3 in each group). Scale bar=50 μm.

One-way ANOVA followed by Tukey’s multiple-comparisons test (B, C, E, F). ***p*<0.01, ns: non-significant.


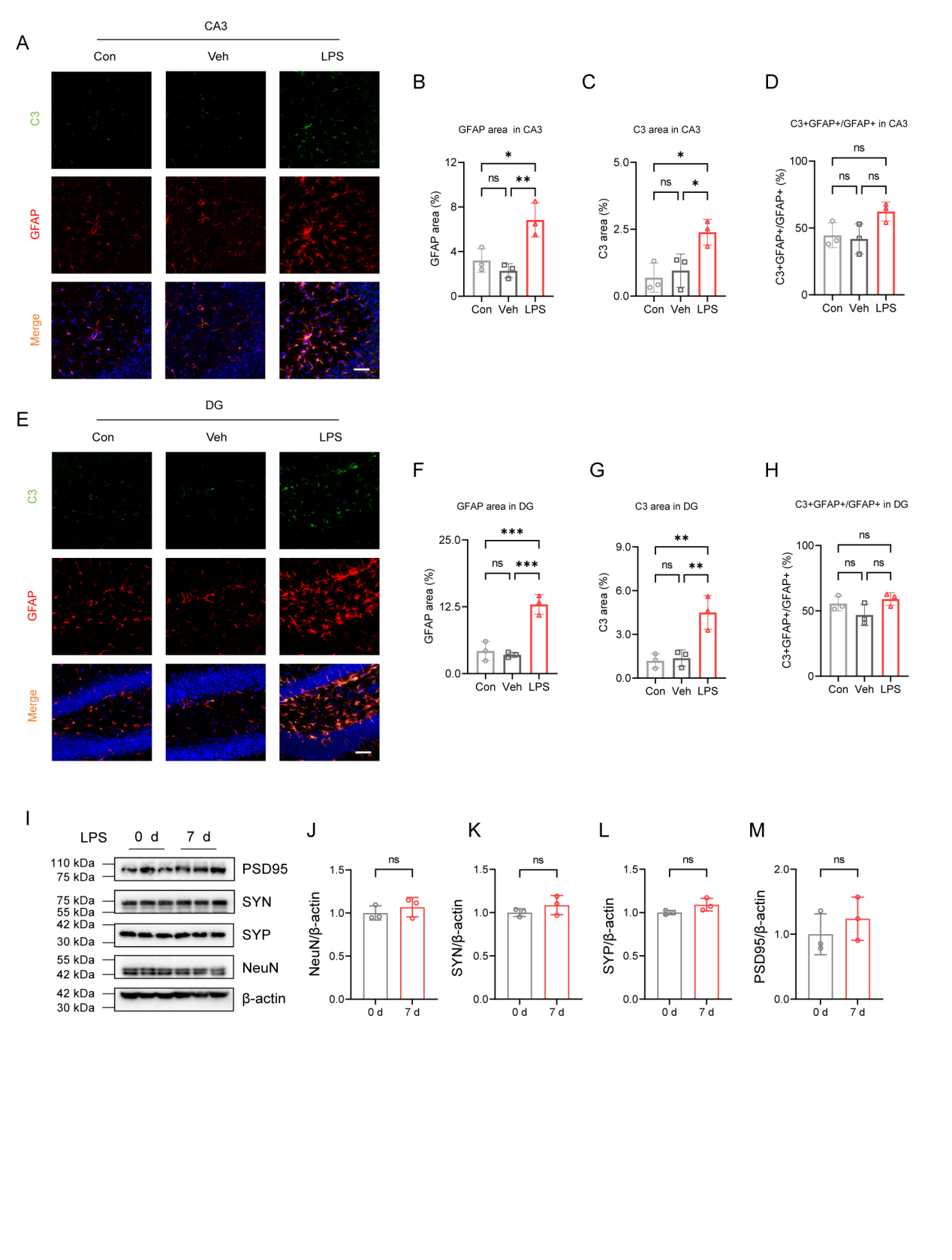


**Fig.S3 LPS administration increases C3 positive astrocytes. Related to Figure 3.**

1. Representative images of C3 (green) and GFAP (red) immunostaining in CA3 (n=3 in each group). Scale bar=50 μm.
2. Quantification of GFAP immunoreactivity area in CA3.
3. Quantification of C3 immunoreactivity area in CA3.
4. Quantification of C3+GFAP+ cells in GFAP+ astrocytes in CA3.
5. Representative images of C3 (green) and GFAP (red) immunostaining in DG (n=3 in each group). Scale bar=50 μm.
6. Quantification of GFAP immunoreactivity area in DG.
7. Quantification of C3 immunoreactivity area in DG.
8. Quantification of C3+GFAP+ cells in GFAP+ astrocytes in DG.

I. Immunoblot analyses of NeuN, SYN, SYP, and PSD95 in the hippocampus.

J-M. Quantification of NeuN (J), SYN (K), SYP (L), PSD95 (M) levels.

One-way ANOVA followed by Tukey’s multiple-comparisons test (B-D, F-H). Unpaired two-tailed student’s *t*-test (J-M). **p*<0.05, ***p*<0.01, ****p*<0.001, ns: non-significant.


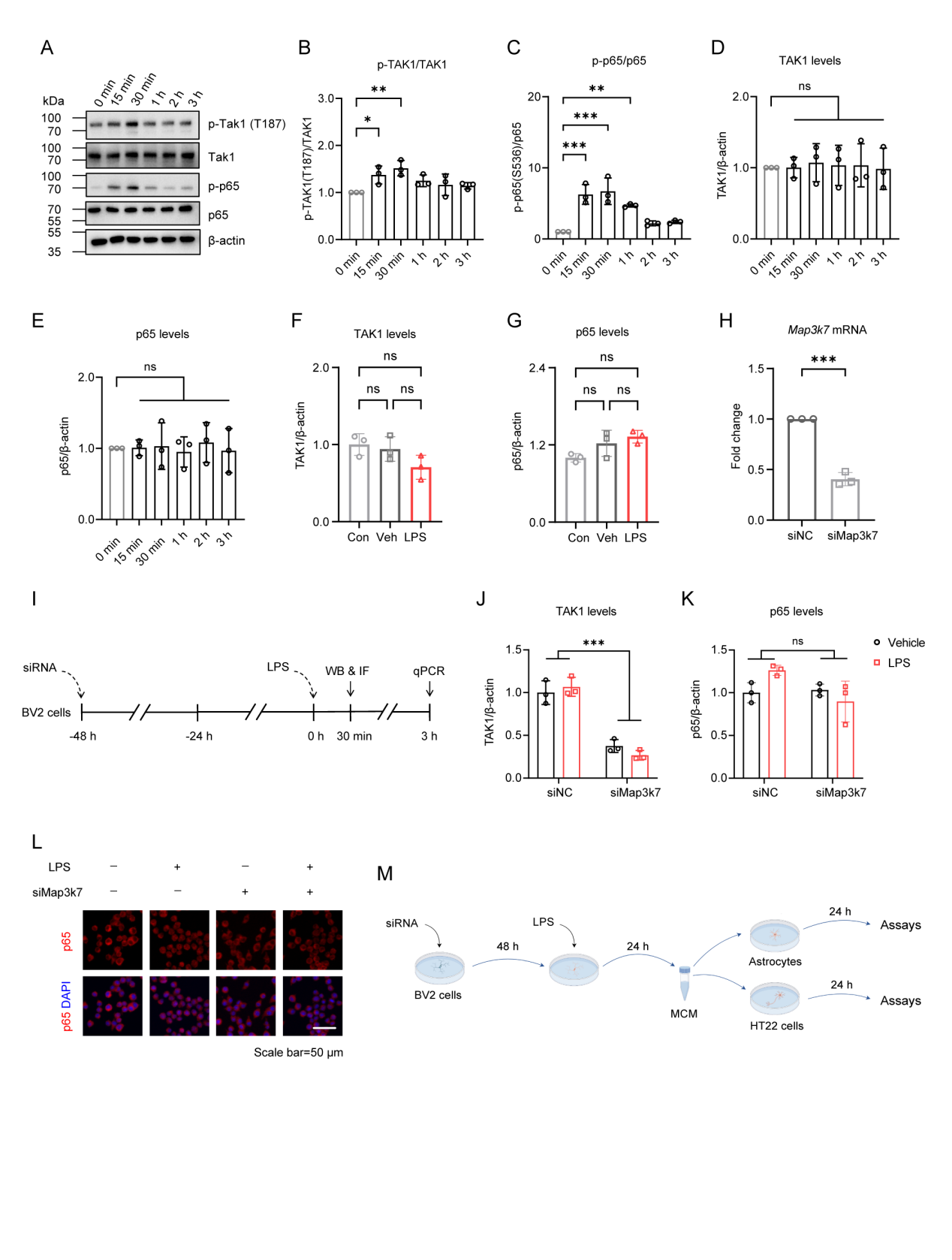


**Fig.S4 TAK1 knockdown inhibits inflammatory responses in BV2 cells. Related to Figure 4.**

1. Immunoblot analyses of p-TAK1 (T187) and p-p65 (S536) in LPS-treated BV2 cells at different time points (n=3 in each group).

B-C. Quantification of p-TAK1 (T187) (B) and p-p65 (S536) (C) at different time points.

D-E. Quantification of TAK1 (D) and p65 (E) at different time points.

F-G. After LPS exposure for 30 min, quantification of TAK1 (F) and p65 (G).

H. Quantification of *Map3k7* mRNA after siRNA treatment in BV2 cells (n=3 in each group).

1. A brief schematic diagram of cellular assays.

J. Quantification of TAK1 in siRNA-treated BV2 cells.

K. Quantification of p65 in siRNA-treated BV2 cells.

L. Immunofluorescence of nuclear p65 in BV2 cells (n=3 in each group). Scale bar=50 μm.

M. A brief schematic diagram of the MCM preparation and the relevant assays.

One-way ANOVA followed by Dunnett’s multiple-comparisons test (B-E). One-way ANOVA followed by Tukey’s multiple-comparisons test (F-G). Unpaired two-tailed student’s *t*-test (H). Two-way ANOVA followed by Tukey's multiple-comparisons test (J, K). **p*<0.05, ***p*<0.01, ****p*<0.001. ns, non-significant.

**
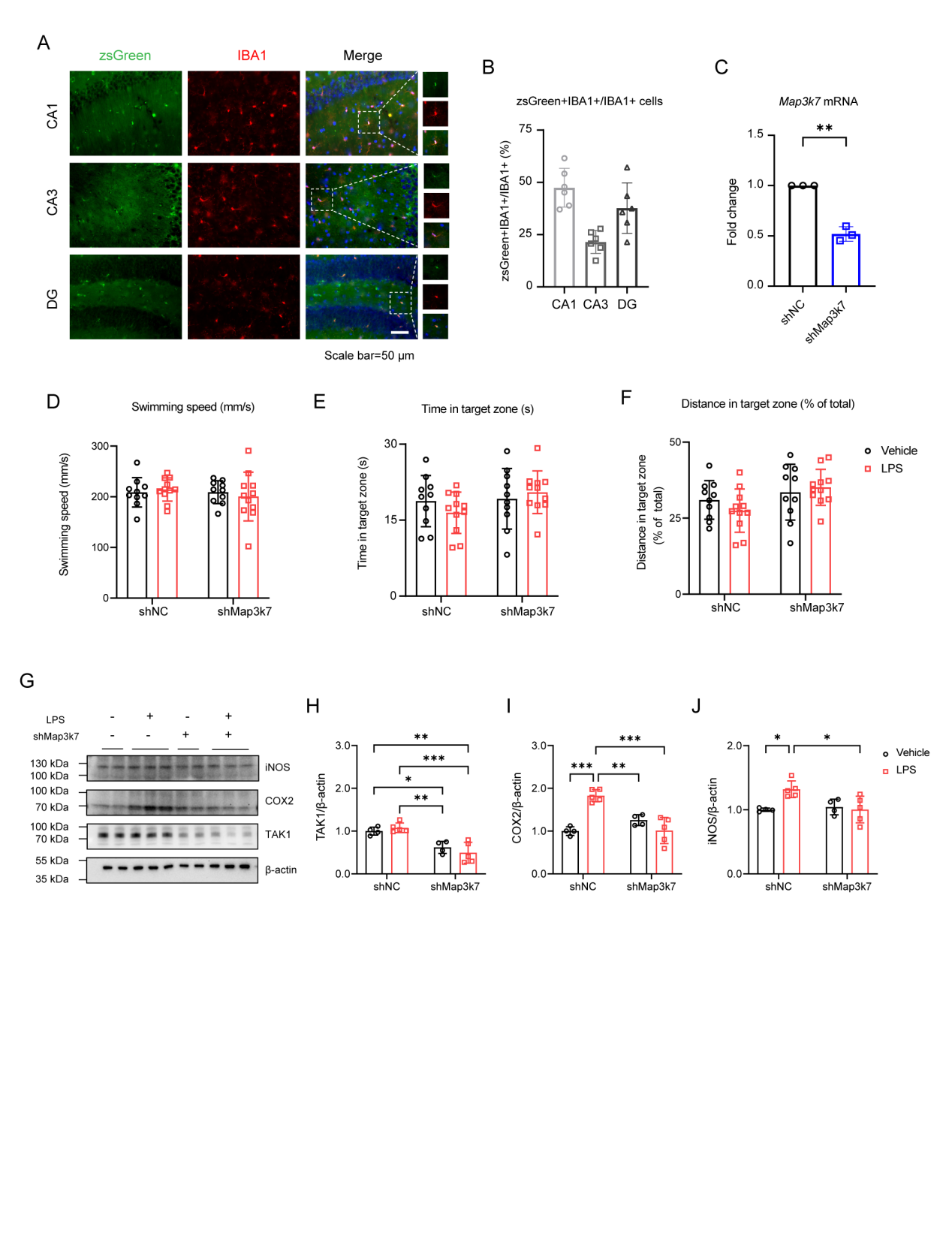
**

**Fig.S5 TAK1 knockdown decreases TAK1, COX2, and iNOS levels in the hippocampus. Related to Figure 6.**

1. Representative images of zsGreen (green) and IBA1 (red) in the hippocampus after injection of AAV-shNC (n=6). Scale bar=50 μm.
2. Proportion of zsGreen+IBA1+ in IBA1+ microglia cells.
3. Quantification of *Map3k7* mRNA after AAV-shMap3k7 injection in mice (n=3 in each group).
4. F. Quantification of swimming speed (D), time (E) and distance (F) in the target zone in probe test (n=10 in Vehicle:shNC and Vehicle:shMap3k7, n=11 in LPS:shNC and LPS:shMap3k7).

G. Immunoblots of TAK1, COX2, and iNOS in the hippocampus (n=4 in Vehicle:shNC and Vehicle:shMap3k7, n=5 in LPS:shNC and LPS:shMap3k7).

H-J. Quantification of TAK1 (H), COX2 (I), and iNOS (J) levels in the hippocampus.

Unpaired *t-*test with Welch’s correction (C). Two-way ANOVA followed by Tukey's multiple-comparisons test (D-F, H-J). **p*<0.05, ***p*<0.01, ****p*<0.001.


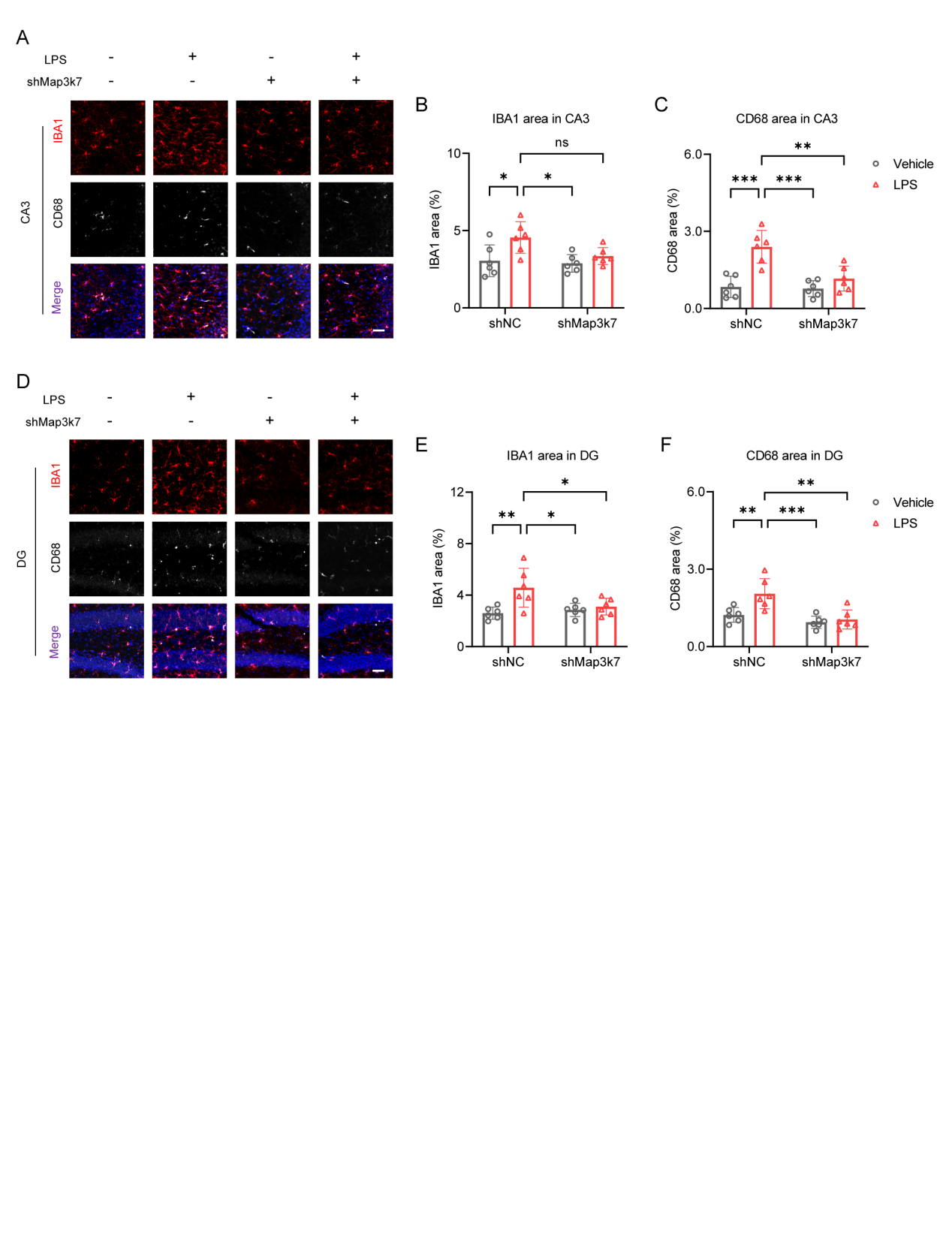


**Fig.S6 TAK1 reduction alleviates microglial activation in the hippocampus. Related to Figure 6.**

1. Representative images of IBA (red) and CD68 (white) immunostaining in CA3 (n=6 in each group). Scale bar=50 μm.

B-C. Quantification of IBA1 (B) and CD68 (C) immunoreactivity area in CA3.

1. Representative images of IBA (red) and CD68 (white) immunostaining in DG (n=6 in each group). Scale bar=50 μm.
2. F. Quantification of IBA1 (E) and CD68 (F) immunoreactivity area in DG.

Two-way ANOVA followed by Tukey's multiple-comparisons test (B, C, E, F). **p*<0.05, ***p*<0.01, ****p*<0.001, ns: non-significant.


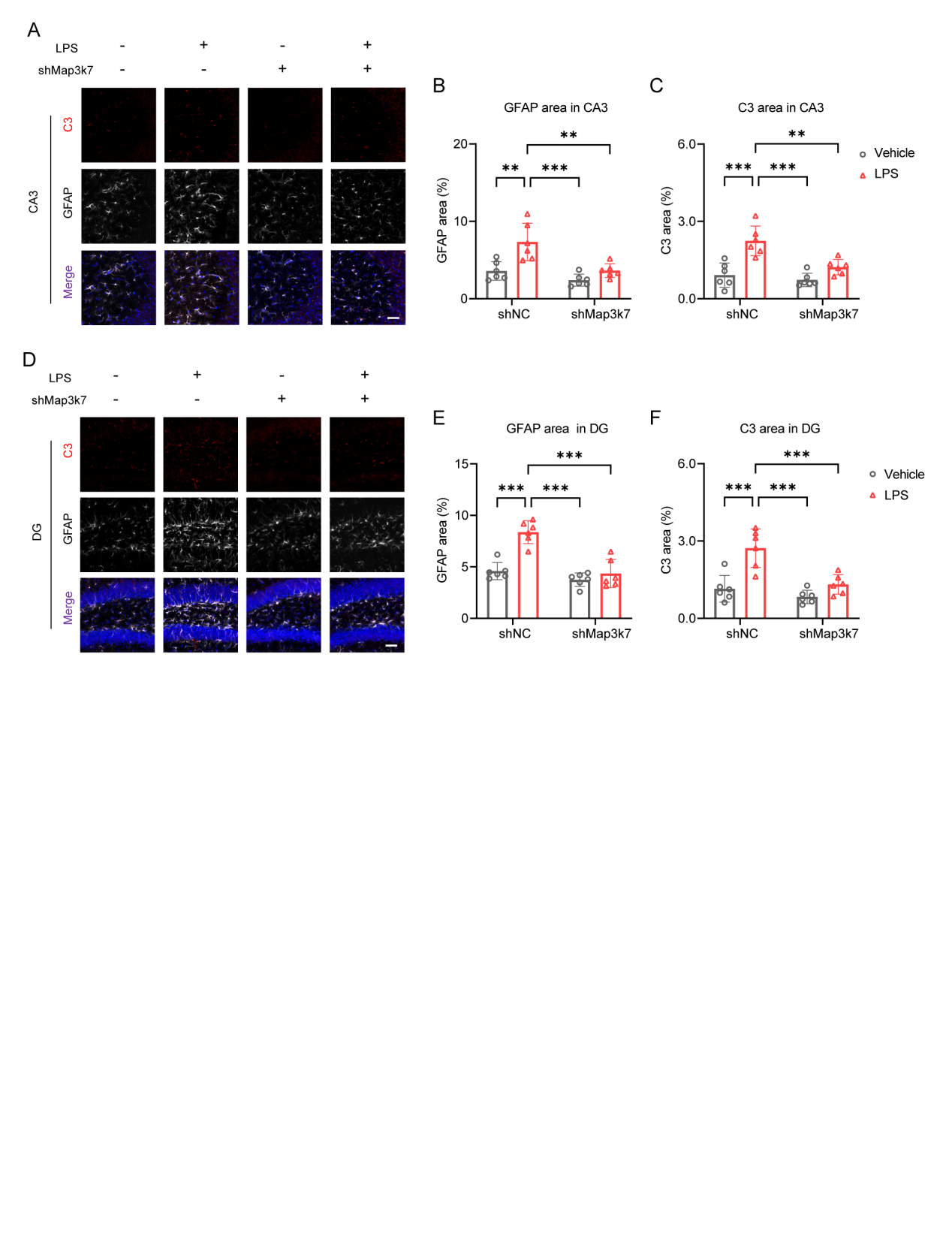


**Fig.S7** **TAK1 reduction limits C3 positive astrocytes in the hippocampus. Related to Figure 7.**

1. Representative images of C3 (red) and GFAP (white) immunostaining in CA3 (n=6 in each group). Scale bar=50 μm.

B-C. Quantification of GFAP (B) and C3 (C) immunoreactivity area in CA3.

D. Representative images of C3 (red) and GFAP (white) immunostaining in DG (n=6 in each group). Scale bar=50 μm.

E-F. Quantification of GFAP (E) and C3 (F) immunoreactivity area in DG.

Two-way ANOVA followed by Tukey's multiple-comparisons test (B, C, E, F). ***p*<0.01, ****p*<0.001.
